# Factors limiting physical activity after acute type A aortic dissection

**DOI:** 10.1007/s00508-018-1412-2

**Published:** 2018-11-19

**Authors:** Thomas Schachner, Fabian Garrido, Nikolaos Bonaros, Christoph Krapf, Julia Dumfarth, Michael Grimm

**Affiliations:** University Clinic of Cardiac Surgery, Innsbruck Medical University, Anichstraße 35, 6020 Innsbruck, Austria

**Keywords:** Aortic dissection type A, Ascending aorta, Sports, Physical activity, Sedentary behavior

## Abstract

**Background:**

Acute type A aortic dissection (AAD) leads to high hospital mortality rates in the first 48 h after the onset of symptoms. Survivors, however, have good long-term perspectives and enhanced survival especially if regaining moderate amounts of physical activity.

**Methods:**

This study analyzed 131 survivors (from 180 consecutive patients, aged 60 years (rande 30–84 years, 71% male) of acute AAD after a median time of 44 months (range 1–147 months). The hospital mortality was 13.5%. The group of physically active patients was compared with those with a sedentary life style. The qualitative and quantitative data on physical activity were correlated with data from an aortic registry.

**Results:**

Overall 87% of patients reported 1 or more types of physical activities after hospital discharge. The most common types were walking (51%), biking (29%), hiking (15%) and gymnastics (14%). Patients with a sedentary life style underwent longer hypothermic circulatory arrest times (39 min, range 8–167 min vs. 47 min, range 27–79 min, *p* = 0.009), had a longer intensive care unit (ICU) stay (Pearsons r = −0.226 [between length of ICU stay and hours of physical activity after hospital discharge], *p* = 0.033) and suffered more frequently from postoperative paresis (33.3% vs. 3.8%, *p* < 0.001) compared with physically active patients. Binary logistic regression analysis showed female gender (*p* = 0.026) and higher body mass index (*p* = 0.019) to be independently associated with a reduced amount of physical activity.

**Conclusions:**

This study demonstrate that the majority of survivors of acute aortic dissection type A regain a physically active life including the practice of a variety of sports. Factors predictive of a sedentary life style can be identified. Female patients deserve special attention.

## Introduction

Acute aortic dissection Stanford type A (involving the ascending aorta) is a fatal disease with a mortality of at least 50% within 14 days that can be reduced to 15–20% by emergency surgery; however, when the patients are discharged long-term survival is encouraging [1, 2]. Many of these patients led an otherwise normal and healthy life prior to the abrupt onset of disease [3]. Therefore, a physically active life should be the target for aortic dissection type A survivors. It is well-established that regular physical activity and aerobic exercise training decrease cardiovascular mortality. The most important benefits for this group of patients are a reduction of blood pressure in hypertensive patients, helping to control body weight, lowering the risk of developing diabetes mellitus, and increasing emotional health [4]. Nevertheless, data on the type and extent of physical and especially sports activities of survivors of acute aortic dissection type A are scarce.

## Methods

In this single institutional follow-up study patients with acute type A aortic dissection after hospital discharge and a median follow-up time of 44 months (range 2–147 months) were included. Physical activity of 5 h or less per week was analyzed by logistic regression for potential sociodemographic and clinical factors. Data were obtained on sports activity from the patients either directly in the outpatient clinic or via telephone interviews. The study included only patients with acute aortic dissection Stanford type A which was operated on as an emergency (in the next available theater). The following information was acquired:

1. Was the patient physically active (sports) preoperatively?
2. Is the patient physically active (sports) postoperatively?
3. Which types of sports, including walking, are performed?
4. What is the amount of sports activity measured in hours per week?

The data were entered into an aortic registry. Approval was obtained from the local ethics committee for the retrospective analysis of risk factors, comorbidities and outcome of the patients with aortic disease in the aortic registry. For statistical analysis, the statistical software package SPSS 17.0 (SPSS, Chicago, IL, USA) was used. Categorical parameters were displayed as numbers and percentages, continuous variables were displayed as median and ranges. Differences between groups were calculated using the Mann-Whitney U-test (continuous variables), bivariate correlation with Pearsons r (continuous variables) and the χ^2^-test (categorical variables). Binary logistic regression analysis was used to identify risk factors for reduced physical activity (<5 h per week). Variables with a *p*-value of 0.1 or less in the univariate analysis were entered into the binary logistic regression analysis. A *p*-value <0.05 was considered statistically significant.

The aim of the study was firstly to survey the amount and type of sports activity of patients who underwent emergency surgery for type A aortic dissection and secondly to define differences between the group of physically active patients and those with a sedentary life style.

## Results

**Fig. 1:**
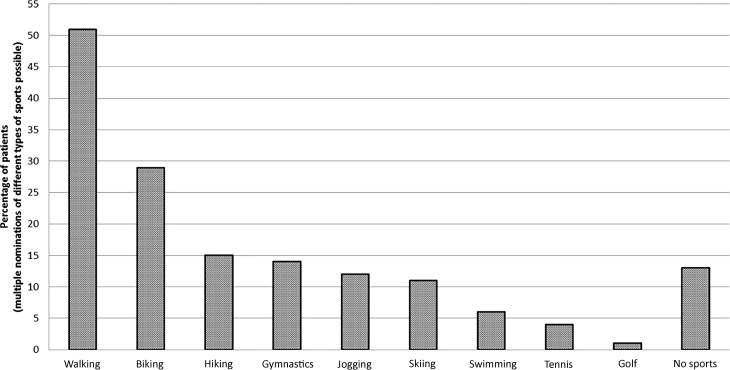
Frequency and types of sports activity of 91 survivors of acute aortic dissection type A and consecutive emergency surgery

**Fig. 2:**
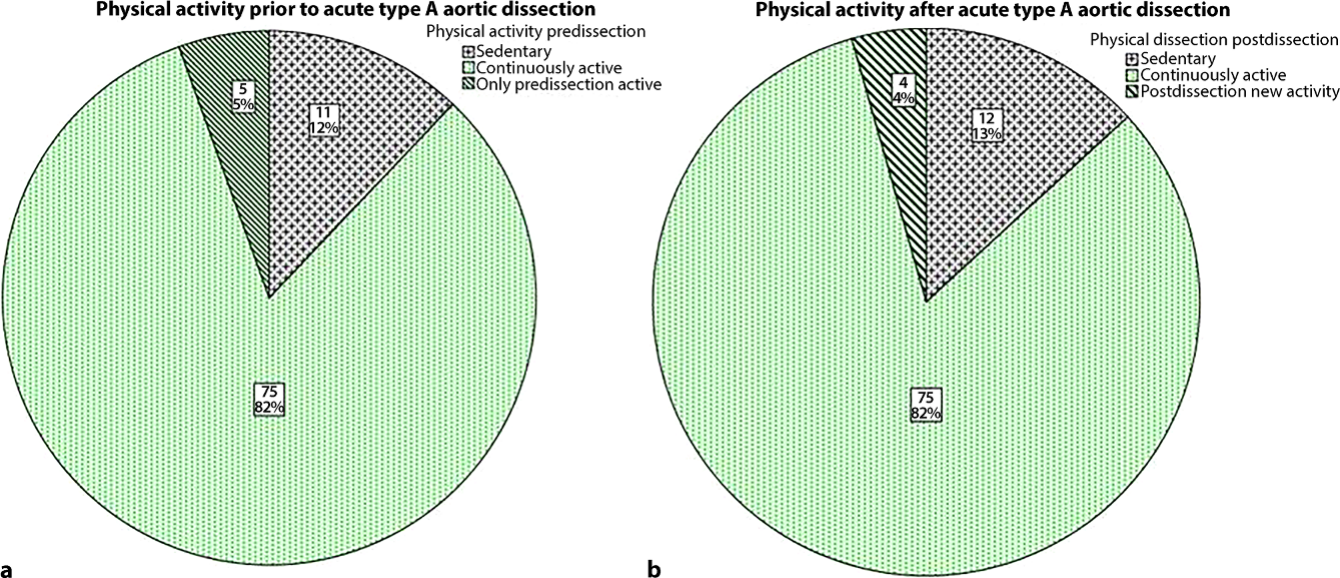
Distribution of physical activity (continuously active, only active predissection or postdissection, sedentary life style). **a** situation prior to the onset of aortic dissection, **b** postoperative situation

From 2001 to 2014 surgery for acute aortic dissection type A was performed on 180 patients. The median age of the patients was 60 years (range 30–84 years) and 71% were male. In total 17 patients were completely lost from follow-up, the majority (12/17) were visitors who lived in foreign countries. Complete data on physical activity could not be obtained from another 30 patients, although survival status was known by patient charts or outpatient visits. From the remaining 133 patients, 42 patients had died at the time of follow-up, resulting in 91 survivors with available data on their sports activity. There was 1 patient with the shortest follow-up of 2 months postoperatively who had already resumed physical activity. A total of 79 out of 91 (87%) patients reported 1 or more sports activities. The frequency and types of sports activity are listed in Fig. 1. The most common types of sports were walking (51%), biking (29%), hiking (15%) and gymnastics (14%). The distribution between continuously active patients and those who stopped or newly began an active life style is shown in Fig. 2.

Tables 1 and 2 show the demography and perioperative data of patients who were physically active compared with patients with a sedentary life style. Patients who were taking exercise had a lower body mass index of 25 (range 17–42) vs. 27 (range 24–38, *p* = 0.027), shorter time from start of aortic dissection symptoms to surgery (370 min, range 128–743 vs. 480 min, range 245–1160 min, *p* = 0.059), shorter hypothermic circulatory arrest times (39 mín, range 8–167 min vs. 47 min, range 27–79 min, *p* = 0.009) and tended to be younger (56 years, range 30–81 years vs. 75 years, range 40–84 years, *p* = 0.09) than those with sedentary life styles.

**Table 1:** Demographic and perioperative data categorized into patients who were physically inactive (sedentary life style) and patients who were physically active

	Sedentary life style (*n* = 12)	Physically active (*n* = 79)	*p*-value
Age, in years (range)	75 (40–84)	56 (30–81)	0.09
Male gender, *n* (in %)	8 (66.7)	59 (74.7)	0.557
Body weight, in kg (range)	85 (64–97)	80 (40–135)	0.089
Body mass index (range)	27 (24–38)	25 (17–42)	0.027
Preoperative serum creatinine, in mg/dl (range)	1.02 (0.63–1.95)	1.0 (0.58–1.90)	0.760
Hypertension, *n* (in %)	10 (83.3)	61 (77.3)	0.802
Diabetes mellitus, *n* (in %)	1 (8.3)	3 (3.8)	0.506
Chronic obstructive pulmonary disease, *n* (in %)	1 (8.3)	4 (5.1)	0.669
Peripheral vascular disease, *n* (in %)	2 (16.7)	12 (15.2)	0.938
Coronary artery disease, *n* (in %)	1 (8.3)	10 (12.7)	0.639
Painless aortic dissection, *n* (in %)	1 (8.3)	3 (3.8)	0.454
Pericardial tamponade, *n* (in %)	1 (8.3)	9 (11.4)	0.732
Preoperative intubated, *n* (in %)	3 (25)	5 (6.3)	0.117
Preoperative neurologic symptoms, *n* (in %)	4 (33.3)	12 (15.2)	0.165
Time from symptom start to operation, in min (range)	480 (245–1160)	370 (128–743)	0.059
Supra-aortic extension of dissection, *n* (in %)	11 (91.7)	57 (72.2)	0.200
Entry tear within ascending aorta, *n* (in %)	7 (58.3)	59 (74.7)	0.270
Cardiopulmonary bypass time, in min (range)	221 (163–600)	204 (109–864)	0.401
Aortic cross clamp time, in min (range)	132 (66–313)	115 (44–337)	0.376
Circulatory arrest time, in min (range)	47 (27–79)	39 (8–167)	0.009
Ventilation time, in h (range)	128 (9–681)	47 (4–902)	0.110
Postoperative renal failure requiring hemofiltration, *n* (in %)	4 (8.9)	11 (13.9)	0.062
Malperfusion, *n* (in %)	3 (25)	5 (6.3)	0.117
Postoperative paresis, *n* (in %)	4 (33.3)	3 (3.8)	<0.001

**Table 2:** Demographic and perioperative data categorized into patients with physical activity (after hospital discharge) up to 5 h per week or more than 5 h per week

	Activity <5 h per week (*n* = 45)	Activity >5 h per week (*n* = 46)	*p*-value
Age, in years (range)	62 (30–84)	56 (30–81)	0.082
Male gender, *n* (in %)	29 (64.4)	38 (82.6)	0.049
Body weight, in kg (range)	80 (45–135)	80 (40–117)	0.881
Body mass index (range)	26 (18–42)	25 (17–36)	0.105
Preoperative serum creatinine, in mg/dl (range)	0.96 (0.58–1.95)	1.00 (0.60–1.90)	0.323
Hypertension, *n* (in %)	34 (75.6)	37 (80.4)	0.951
Diabetes mellitus, *n* (in %)	3 (6.7)	1 (2.2)	0.273
Chronic obstructive pulmonary disease, *n* (in %)	4 (8.9)	1 (2.2)	0.137
Peripheral vascular disease, *n* (in %)	9 (20)	5 (10.9)	0.176
Coronary artery disease, *n* (in %)	6 (13.3)	5 (10.9)	0.628
Painless aortic dissection, *n* (in %)	1 (2.2)	3 (6.5)	0.306
Pericardial tamponade, *n* (in %)	4 (8.9)	6 (13.1)	0.577
Preoperative intubated, *n* (in %)	6 (13.3)	2 (4.4)	0.288
Preoperative neurologic symptoms, *n* (in %)	12 (26.7)	4 (8.7)	0.023
Time from symptom start to operation, in min (range)	435 (155–1160)	351 (128–743)	0.397
Supra-aortic extension of dissection, *n* (in %)	35 (77.8)	33 (71.8)	0.611
Entry tear within ascending aorta, *n* (in %)	34 (75.6)	32 (69.6)	0.905
Cardiopulmonary bypass time, in min (range)	199 (109–600)	208 (138–864)	0.239
Aortic cross clamp time, in min (range)	107 (45–313)	129 (44–337)	0.448
Circulatory arrest time, in min (range)	42 (8–79)	38 (22–167)	0.634
Ventilation time, in h (range)	51 (7–744)	43 (4–902)	0.174
Postoperative renal failure requiring hemofiltration, *n* (in %)	8 (17.8)	7 (15.2)	0.942
Malperfusion, *n* (in %)	6 (13.3)	2 (4.4)	0.078
Postoperative paresis, *n* (in %)	6 (13.3)	1 (2.2)	0.061

*h* hour

**Fig. 3:**
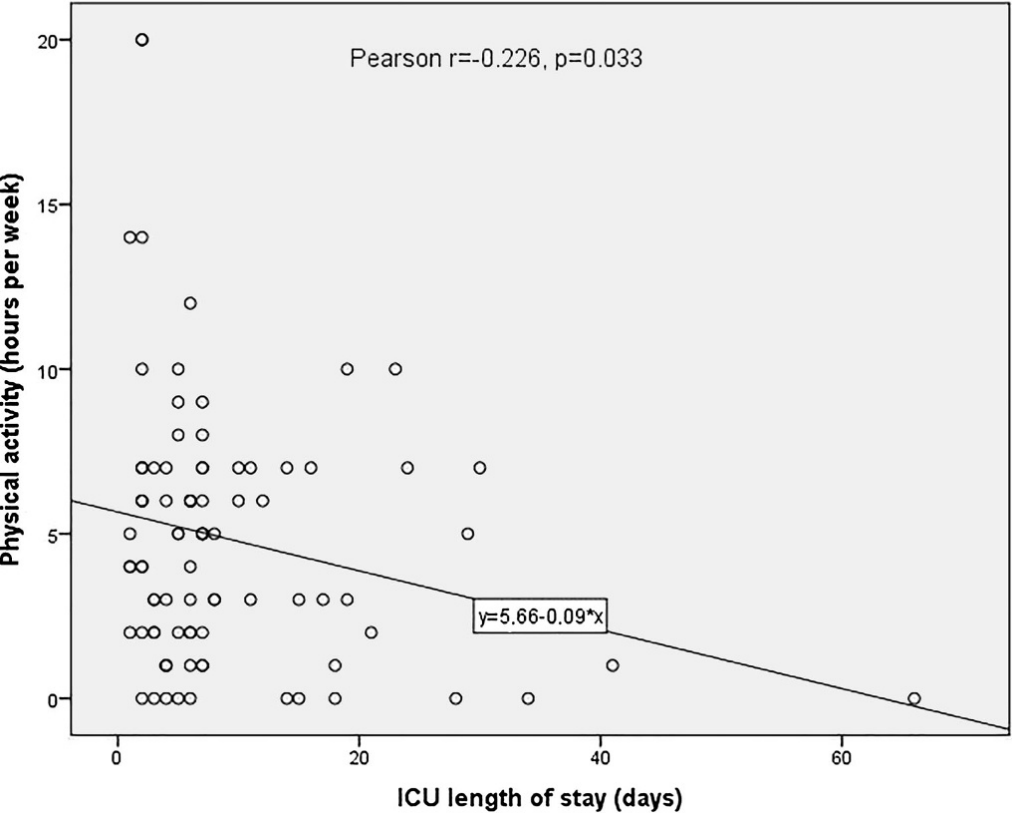
Bivariate correlation between length of stay at intensive care unit (ICU) and hours of physical activity per week

The length of stay in the intensive care unit correlated significantly with the amount of physical activity (hours per week, Pearsons r = −0.226, *p* = 0.033, Fig. 3).

In addition, postoperative renal failure requiring hemofiltration (13.9% vs. 8.9%, *p* = 0.062) and postoperative paresis (33.3% vs. 3.8%, *p* < 0.001) were more frequently found in patients with a sedentary life style compared with the active group of patients. The sports activity was quantified with a median amount of 5 h (1–20 h) per week. Male gender 82.6% vs. 64.4%, (*p* = 0.049), and a trend towards younger age (56 years, range 30–81 years vs. 62 years, range 30–84 years, *p* = 0.082), was more frequently found in the group which completed more than 5 h sports per week compared with the less active group. In contrast, preoperative neurologic symptoms (26.7% vs. 8.7%, *p* = 0.023), malperfusion (13.3% vs. 4.4%, *p* = 0.078), and postoperative persistent paresis (13.3% vs. 2.2%, *p* = 0.061) was more often found in the group which practiced sports less than 5 h per week.

The initial symptoms of aortic dissection were chest pain in 79%, other pain (abdominal, neck, lower extremity pain) in 11%, and painless presentation in 4%. Painless aortic dissection was not associated with physical activity (Table 1 and 2). Binary logistic regression analysis showed that a higher body mass index (*p* = 0.030), increasing age (*p* = 0.035) and female gender (*p* = 0.018) were independently associated with a lower amount of physical activity (5 h or less per week, Table 3).

**Table 3:** Binary logistic regression analysis of variables associated with a lower amount of physical activity (5 h or less per week)

	Wald	Sig.	Exp (B)	95% CI for Exp (B)	
Male gender	5.552	0.018	0.056	0.005	0.615
Age (years)	4.462	0.035	1.102	1.007	1.205
Body mass index	4.734	0.030	1.294	1.026	1.633
Preoperative neurologic symptoms	0.526	0.468	5.113	0.062	419.667
Postoperative paresis	0.011	0.916	1.305	0.009	184.869
Postoperative malperfusion	3.199	0.074	20.875	0.747	583.006

*Wald, Sig. Exp (B), CI* confidence interval, *h* hour

## Discussion

Patients who have survived the life-threatening event of aortic dissection are confronted with the question to what extent they can regain full physical activity including sports. Not only the variably extensive replacement of the thoracic aorta and aortic valve but also the presence of a lifelong aortic dissection of the descending aorta in most of the patients results in a sudden loss of confidence in doing sports. Acute aortic dissection often affects otherwise healthy and physically active people. Hence regaining a physically active life is not only important for physical and emotional health but also the patients desire to get back at least in part into the old way of life. This is impressively reflected by these data: Almost 9 out of 10 patients participated in regular physical activity. This distribution is similar between pre-aortic and post-aortic dissection, with approximately 5% of patients either stopping activity or becoming newly active due to the emergency event. The rate of patients doing sports after aortic dissection type A is comparable with the rate of people who are doing any sports in Austria. In a study by Pratscher this rate was 67% [5]. Chaddha et al. reported a rate of physical activity of 76% in a mixed cohort of 82 patients with either type A (*n* = 45) or type B aortic dissection [6].

The recommendations for sports activity after aortic dissection focus on prevention of high peaks of blood pressure. The patient is generally advised to avoid maximum exertion of weightlifting or sprinting and certain activities, such as wood chopping, shovelling snow and mowing the lawn with a non-self-propelled mower [7]. Typically, patients are also advised to avoid competitive sports and contact sports; however, recommendations become more vague when it comes to commonly performed physical activities. It is helpful to use a table of metabolic equivalents to classify different types of physical activity. The reference activity with a metabolic equivalent (ME) of 1.0, is defined by a resting position (lying quietly). Since systolic blood pressure increases with every rise of the ME patients should not choose sports in the high range zone. The calculated metabolic equivalents of the sports activities found in this study were: walking slowly (<2 miles per hour, i.e. <3.2 km/h): 2.0, walking briskly (3 mph, i.e. 4.8 km/h): 3.3, bicycling (casual, <10 mph, i.e. <16.1 km/h): 4.0, strenuous hiking: 6.0–7.0, jogging: 10.2, downhill skiing: 6.8, swimming at a slow pace: 4.5, tennis (doubles): 5.0, tennis (singles): 7.0–12.0 and golf (without cart, carrying heavy bag of clubs): 4.4. Interestingly, the sports activities of this patient cohort covered a wide range of ME groups.

In this study younger patients did more sports than older patients. This is in agreement with the general Austrian population, where people aged >70 years showed a markedly reduced rate of sports activity compared with the younger population [5]. Another interesting result of this study was that in patients who did sports the time from symptom start to diagnosis was almost 2h shorter compared with the sedentary life style group. The International Registry of Acute Aortic Dissection Investigators found that patients with delayed time to operation more frequently presented with atypical symptoms [8]. An important subgroup are patients with painless aortic dissection. Imamura et al. demonstrated that patients with painless aortic dissection more frequently suffered from cerebrovascular disease. The same study found worse functional outcome at discharge as evaluated with overall performance category (OPC) [9]. These factors could explain reduced sports activity in this group of patients.

This study clearly showed that both longer hypothermic circulatory arrest times and a higher rate of postoperative paresis were found in the less active group of patients. An explanation for this is that longer circulatory arrest times in aortic surgery are a surrogate for a more complex disease of the aortic arch. Furthermore, hypothermic circulatory arrest leads to a certain amount of organ ischemia despite the use of selective cerebral perfusion. The negative impact of neurological injury on physical activity is obvious. Krähenbühl et al. demonstrated that even transient neurological dysfunction after aortic surgery impaired quality of life using the SF36 quesionnaire [10].

In addition, Bashour et al. found that longer intensive care unit (ICU) stays (>10 days) after cardiac surgery were associated with reduced survival as well as a low Duke activity score index of 26. This is in agreement with the findings of this study of a significant inverse relationship between length of ICU stay and hours of physical activity per week [11]. Interestingly female gender emerged as a risk factor for a decreased amount of physical activity in this study. In a recently published survey among working people aged 30–64 years in Germany, women less frequently paerformed physically activity >2.5 h per week than men (41% vs. 45%, *p* < 0.001) [12].

### Limitations of the study

This is a retrospective study and data on physical activity of deceased patients were not available. It could be speculated that the fitness of patients with a decreased survival rate might be inferior compared with those patients with a higher postoperative life expectancy. A second limitation is the fact that physical activity was quantified by self-report with an inherent subjectivity.

## Conclusion

A sedentary life style is a growing global epidemic with a huge impact on public health. This study demonstrated that the majority of survivors of acute aortic dissection type A resume a physically active life. A variety of sports activities is practiced and only a few patients stopped physical activity due to this life-threatening disease. The study identified patients at risk, especially female patients, for decreased physical activity for which it might be useful to offer special rehabilitation therapy.
